# Validation of a Worst-Case Scenario Method Adapted to the Healthcare Environment for Testing the Antibacterial Effect of Brass Surfaces and Implementation on Hospital Antibiotic-Resistant Strains

**DOI:** 10.3390/antibiotics9050245

**Published:** 2020-05-12

**Authors:** Emilie Dauvergne, Corinne Lacquemant, Crespin Adjidé, Catherine Mullié

**Affiliations:** 1 Laboratoire AGIR - UR UPJV 4294, UFR de Pharmacie, Université de Picardie Jules Verne, 80037 Amiens, France; emilie.dauvergne7@laposte.net; 2 FAVI Limited Company, 80490 Hallencourt, France; clacquemant@favi.com; 3 Laboratoire Hygiène, Risque Biologique et Environnement, CHU Amiens-Picardie, 80025 Amiens, France; crespin.adjide@chu-amiens.fr

**Keywords:** copper, brass, antibacterial efficiency, hospital acquired infections, antibiotic resistance, antibacterial surfaces

## Abstract

The evaluation of antibacterial activity of metal surfaces can be carried out using various published guidelines which do not always agree with each other on technical conditions and result interpretation. Moreover, these technical conditions are sometimes remote from real-life ones, especially those found in health-care facilities, and do not include a variety of antibiotic-resistant strains. A worst-case scenario protocol adapted from published guidelines was validated on two reference strains (*Staphylococcus aureus* ATCC 6538 and *Enterobacter aerogenes* ATCC 13048). This protocol was designed to be as close as possible to a healthcare facility environment, including a much shorter exposure-time than the one recommended in guidelines, and evaluated the impact of parameters such as the method used to prepare inocula, seed on the surface, and recover bacteria following exposure. It was applied to a panel of 12 antibiotic-resistant strains (methicillin resistant, vancomycin-resistant, beta-lactamase, and carbapenemase producing strains as well as efflux pump-overexpressing ones) chosen as representative of the main bacteria causing hospital acquired infections. Within a 5-min exposure time, the tested brass surface displayed an antibacterial effect meeting a reduction cut-off of 99% compared to stainless steel, whatever the resistance mechanism harbored by the bacteria.

## 1. Introduction

Hospital acquired infections (HAIs) are a major public health issue worldwide [1] and several preventive measures are currently used to limit them, including biocleaning, awareness campaigns, and hospital hygiene procedures, notably the use of hydroalcoholic hand-rubs [2]. Hospital environments also play a crucial role in HAIs [3,4,5]. Of the four means of transmission identified for HAIs, contaminated hands and/or surfaces account for up to 20–40% of pathogen transmission [6,7,8]. One of the preventive measures envisaged to reduce the burden of HAIs is to equip healthcare facilities with contact surfaces composed of antimicrobial materials such as copper alloys or antibacterial plastics. This would help in continuously reducing the presence or persistence of microorganisms on surfaces (as opposed to biocleaning actions the effect of which is temporary) and thus in indirectly lowering their diffusion [9,10].

Copper and copper alloys used to reduce the bioburden on surfaces were tested on several bacteria that could be found in the hospital environment such as *Escherichia coli* [11]. However, some of the bacteria causing HAIs can display natural and/or acquired resistance to antibiotics, disinfectants/detergents, and/or antimicrobial surface components such as copper. Several copper-resistance mechanisms have previously been reported in the agri-food sector and water environment [12]. These mechanisms have been correlated to disinfectant and antibiotic resistance mechanisms through co-selection, co-resistance, and cross-resistance [13]. For example, *tcrB* gene is linked to genes encoding macrolide and glycopeptide resistances [14], *oqx*AB co-exists with the production of beta-lactamases and *pco* resistance operons, and *mcr*-1 was detected in copper-tolerant isolates [15]. Therefore, it appears important for antibacterial materials to check for the absence of combined antibiotic resistance with typically isolated antibiotic-resistant bacterial strains found in healthcare facilities.

Prior to trials in those facilities, a multitude of protocols has been designed to first demonstrate the in vitro antibacterial effectiveness of copper alloys [11,16,17,18]. However, few technical justifications supporting the choice of key parameters included in these protocols are available. The impact of several of these parameters has not so far been systematically reported, including (i) the number of subcultures prior to the assay, (ii) the sample size, (iii) the cleaning and disinfection of sample before the assay, (iv) the quantity and volume of the inoculum deposited, (v) spreading or not spreading the inoculum over the surface, (vi) the parameters for taking into account the drying time, (vii) the volume and composition of the recovery fluid, (viii) the recovery technique, and (ix) the volume of recovery fluid taken and the dilutions used to carry out the enumerations. Additionally, different exposures have been reported in published protocols and could affect the survival of microorganisms on the surfaces and the resulting efficacy of the antimicrobial surface [19]. Nevertheless, efforts to standardize these laboratory-testing conditions for non-porous surfaces have been made to grant an “antimicrobial” label for non-porous materials such as copper alloys [20,21,22,23]. Although these protocols allow to compare the effectiveness of copper alloys with a standardized method, they use bacterial strains that have resistance levels and conditions that are not close enough to real-life ones.

Therefore, our goal was to develop a standardized protocol to test the antibacterial properties of copper alloys establishing a worst-case scenario (WCS) close to real-life conditions. Once the optimal parameters for the WCS were set, the protocol was deployed on 12 clinical strains representative of the major pathogen agents isolated from nosocomial infections and of various antibiotic-resistance mechanisms to ascertain that the antibacterial effect of the copper alloy was not impaired by these mechanisms.

## 2. Results

### 2.1. Normalization of the Inoculum

Prior to the sample inoculation, no significant differences were observed for inoculum counts, whatever the number of subcultures (two or three) or incubation time (24 or 48 h) (Table 1). Post-inoculation of the samples, significant differences were found for the bacterial recovery on brass. Bacterial recovery was lower when the inoculum was prepared with a strain subcultured for 48 h three times as compared to only twice. Similarly, bacterial recovery was lower when the inoculum was prepared with a strain subcultured for 24 h three times as compared to twice (Table 1).

### 2.2. Spread vs. Non-Spread Inoculum

The bacterial recovery obtained with a 9 µL spread inoculum was significantly lower than with a 1 µL non-spread one (Table 1).

### 2.3. Bacterial Recovery Technique Following Exposure to Metal Surfaces

The results between the neutralizing volumes of 10 or 20 mL of Letheen broth showed no significant differences (Table 1, *p* = 0.66, Mann–Whitney test). Comparison between glass beads and ultrasonication as recovery techniques did not uncover significant differences either (Table 1, *p* = 0.76, Mann–Whitney test).

### 2.4. Setting of WCS Parameters and Validation on Two Reference Strains

From the results obtained above, the following settings were chosen for the WCS protocol: an inoculum prepared with a strain subcultured twice for 24 h, a non-spread deposit of 1 µL, a Letheen broth recovery volume of 10 mL and ultrasonication as a recovery technique for surviving bacteria. The exposure-time was reduced to a minimum (5 min, typically).

Two antibiotic-susceptible bacterial strains, *Enterobacter aerogenes* ATCC13048 and *Staphylococcus aureus* ATCC6538, referenced in US EPA and/or AFNOR guidelines, were used to validate the WCS protocol [22,23]. Statistically significant reductions were observed between the brass alloy and stainless steel for both strains. The calculated reduction percentages were 99.97% ± 0.017% and 99.97% ± 0.004% for *S. aureus* ATCC6538 and *E. aerogenes* ATCC13048, respectively (*p*-values < 0.0001, Mann–Whitney test). These results are consistent with the recommendations for efficacy of non-porous antimicrobial surfaces of both AFNOR and US EPA (99% and 99.9% of reduction between brass and stainless steel, respectively) [22,23] and validate a bactericidal effect on Gram negative and positive strains for the tested alloy. The WCS protocol was therefore approved and used as such for the following experiments.

### 2.5. Deployment of the WCS Protocol on 12 Antibiotic Resistant Strains of Bacteria

A panel of antibiotic-resistant strains was tested with the WCS method validated above. This panel consisted of four positive Gram and eight negative Gram strains representing bacteria the more frequently responsible for HAIs and HAI outbreaks (Table 2).

#### 2.5.1. Gram Positive Bacteria

Among the four Gram positive strains tested, both SAAM 13 and SAAM 118 showed an efficient reduction on the brass and copper surfaces compared to stainless steel (Table 3). While brass and pure copper both met the Agence Française de Normalisation (AFNOR) 99% reduction criterion for SAAM 33 and SAAM 118, the reduction was significantly better for copper as compared to brass for SAAM 33. This was not the case for SAAM118 (Table 3). EFISAM2 and EFUMAM2 were selected as representatives of Vancomycin-Resistant Enterococci (VRE) and harbored *vanB* and *vanA* genes, respectively. On the one hand, EFISAM2 proved to be an unsuitable strain for the implementation of the WCS protocol as it was not recovered on any of the tested surfaces, including stainless steel. On the other hand, EFUMAM2 inoculum was significantly reduced on both brass and copper compared to stainless steel (Table 3).

#### 2.5.2. Gram Negative Bacteria

Four of the eight Gram-negative strains tested in this work belonged to the Enterobacteriaceae family. Compared to 304 L stainless steel, a significant reduction of inocula was witnessed for all four strains on the brass and pure copper surfaces, whatever the resistance mechanism(s) (Table 3). As for the four remaining Gram-negative strains, they belonged to the non-lactose fermenting category. They consisted of two *A. baumannii* and two *P. aeruginosa* strains. ABAM14 could not be recovered from any of the surfaces tested while both the brass and copper surfaces generated a significant reduction of ABAM41 inoculum compared to 304L stainless steel (Table 3). *P. aeruginosa* AM85 and PAAM10 saw their inocula significantly reduced by either the brass or the copper surfaces (Table 3).

## 3. Discussion

The results obtained for the normalization of the inoculum with McFarland standards proved that it was efficient. This step appeared as mandatory because inocula prepared directly from bacteria grown in Luria broth in accordance with United States Environmental Protection Agency (US EPA) guidelines generated non-reproducible inoculum counts (data not shown). Variable inocula can lead to fluctuating end-results in bacterial reductions, just like minimum inhibitory concentration (MIC) determinations are liable to variations depending on the inoculum effect [24]. A standardization of the incoulum should be carried out systematically to ensure maximum reproducibility of the results. The results obtained by comparing the number of subcultures and incubation time on the recovery of the strain on brass highlight the impact of these parameters on antibacterial efficiency results. They should therefore be taken into account. In the literature, few arguments on the evaluation of the impact of incubation time or/and the number of subcultures for inocula preparation could be found for non-porous surfaces antibacterial efficacy tests [11,25]. Regarding the incubation time, recommendations from the AFNOR (three subcultures for 24 h) and the US EPA (three subcultures for 48 h) are not in agreement for the inoculum preparation [22,23]. In this study, recovery results on brass samples were the highest (worst-case scenario) with two subcultures of a strain incubated for either 24 or 48 h. As the enumerations were similar whatever the subculture number, the choice of two subcultures was made in order to remain close to the genetic profile of the reference strain and avoid too much genetic variability. The following assays were therefore performed with inocula prepared with a strain subcultured twice for 24 h. Nevertheless, it has to be pointed out that previous works have shown that using a strain subcultured once for 24 h also gave consistent results and would allow for the minimal genetic variation [11].

The 9 µL spread and 1 µL non-spread deposit conditions were compared because they were adapted from the US EPA (9 µL instead of 20 µL to take the sample surface into account) and AFNOR (1 µL) ones [22,23]. In both methods, smearing at 3 mm from the edge of samples was performed. The smearing procedure is typically used to limit the surface tension, which depends on physical and chemical proprieties of the said surface [26]. However, a non-spread 1 µL inoculum was chosen in this study because it was more representative of the hospital environment reality in terms of volume and contact with the surface for contaminating droplets such as with saliva or sink ones, for example [27], and represents the worst case situation of both deposit conditions compared here.

Standardized protocols propose different volumes of neutralizing solution and bacterial recovery techniques (10 mL and ultrasonication for AFNOR vs. 20 mL with glass beads agitation for US EPA) [22,23]. Ultrasonication has been reported to induce the degradation of the bacterial cell wall leading to a lower recovery of the surviving bacteria [28]. However, the results from our experiments did not confirm this point (Table 1). Moreover, the smallest volume used in AFNOR protocol mechanically allows for a detection limit twice lower than that of the EPA protocol. Therefore, the choice was made to keep a volume of 10 mL Letheen broth along with ultrasonication for the recovery of surviving bacteria on metal samples.

To ascertain than the new brass alloy and copper retained their activity against antibiotic-resistant strains that are more likely to be found on surfaces in healthcare facilities, a panel of 12 strains was selected with varying resistance mechanisms, some of which have previously been linked with copper resistance. Both methicillin-resistant *S. aureus* (MRSA) were also resistant to fluoroquinolones. In addition, SAAM 33 was not susceptible to aminoglycosides. Whatever the resistance profile, the brass and copper surfaces were highly efficient. The same conclusion could not be reached for enterococci as EFISAM2 could not be recovered from any of the surfaces tested. This strain was thus hypothesized to be highly susceptible to desiccation in our test conditions. On the other hand, EFUMAM2 inoculum was significantly reduced on both brass and copper surfaces compared to the stainless steel one. Links have been established between copper and glycopeptides resistances in VRE strains [13,14]. However, the susceptibility to desiccation of the *van*B bearing strain precluded the validation of the brass and copper surfaces efficiency on these kinds of strains. These results emphasize the differences in behavior between strains of a single species and advocate for a systematic validation of the strains chosen before implementing large scale assays.

*Enterobacteriaceae* strains included in this work were representative of Extended-Spectrum β-Lactamase (ESBL) and carbapenemase producing strains, very common among bacteria responsible for HAIs [29,30,31,32]. Whatever the resistance mechanism displayed by these strains, the antibacterial efficiency of brass and copper surfaces was not impaired, even with the short exposure time used in this WCS protocol.

Carbapenem-resistant *A. baumannii* (CRAB) strains were chosen because they have been identified as an increasing threat worldwide, especially in intensive care units (ICU) [33]. No reduction could be calculated for ABAM14 as it could not be recovered from the stainless steel surface. No conclusion could therefore be drawn for this strain apart from its susceptibility to desiccation. This finding was unexpected as *A. baumannii* species is usually described as being able to survive in the environment for long periods of time. However, this characteristic appears to be highly dependent on the strain [34], once more pointing out behavioral discrepancies between two strains of a single species. The second CRAB strain, ABAM41, was isolated from the surface of a stainless steel trolley of an ICU of Amiens hospital, vouching for its resistance to desiccation. It was classified as an extremely drug resistant (XDR) strain because of its resistance to most antibiotic classes including β-lactams, aminoglycosides, and fluoroquinolones. ABAM 41 was included in the panel because its sampling site was an ideal location for an indirect transmission of a nosocomial infection. Despite its XDR profile, the antibacterial efficiency of brass on this strain met the US EPA reduction cut-off of 99.9% after a 5-min exposure. This result highlights the benefit of using brass surfaces to help in reducing surface-transmitted XDR *A. baumannii* strains. As for *P. aeruginosa* strains, the results obtained for carbapenemase (VIM)-producing PAAM10 was interesting as a link has previously been described between *P. aeruginosa* ST308 strains which can produce β-lactamases such as VIM-2 and resistance to copper [12]. Similarly, AM85 was previously shown to overexpress *mexB*, *mexF*, and *mexY* genes coding for components of efflux pumps belonging to the Resistance-Nodulation-Division (RND) family [35]. RND efflux pumps have also been pointed out as contributing to copper resistance in Gram-negative bacteria [36]. It is therefore of interest to validate the efficacy of copper and brass surfaces on such strains.

Overall, the brass surface displayed a bactericidal effect in accordance with the 99% efficacy recommendation for non-porous antimicrobial surfaces of AFNOR for 10 out of the 12 strains tested (Table 3). Additionally, the brass alloy also met US EPA recommendations (99.9% reduction) for ABAM 41, EFUMAM2, AM85, and PAAM10. These reductions were obtained following a short (5-min) exposure timeframe when exposure times in existing guidelines vary between 1 and 2 h. This finding shows that the bactericidal activity is obtained quickly on brass which is important if the goal is to prevent surface-transmitted contaminations. The pure copper surface used as positive control gave results complying with both AFNOR and US EPA recommendations for five strains (KPNAM1, AM85, PAAM10, SAAM33, and SAAM118) while ECLOAM1 had a reduction only consistent with the 99% reduction recommended by AFNOR (Table 3). For two remaining strains (ECLOAM1 and SAAM33), a significantly better reduction was witnessed on copper than on brass (Table 3). It has previously been shown that the efficacy of copper alloy surfaces depended on the amount of copper in the final alloy [11]. The higher the percent of copper is and the more efficient the bactericidal effect is [11,37]. This was not systematically the case here (e.g., EFUMAM2 and ECLOAM1). The hypothesis put forward to explain this discrepancy could be a different reaction to the surface treatment for pure copper compared to brass, reducing the pure copper efficacy [38]. Oxidization reactions might have developed at a different pace and more unevenly on the treated copper surface than on the brass one. This could account for the important variations witnessed for some strains (e.g., EFUMAM2) for one or more of the three samples tested in a series of experiments, leading to some high standard deviation values.

## 4. Materials and Methods 

All tests were conducted at least three times on a minimum of three samples.

### 4.1. Metal Samples

Three types of metal samples were used in this study: 304L stainless steel (negative control of antimicrobial activity), pure copper (99.95%) (positive control of antimicrobial activity), and a brass alloy (62.2% of copper) (FAVI, Hallencourt, France). The sample size was 18.05 × 19.93 mm. Brass samples were produced using the die-casting foundry process. Copper and stainless steel samples were obtained by laser cutting from sheets. All samples underwent the same surface treatment prior to use.

### 4.2. Sample Preparation

Prior to the assay, all samples were cleaned with acetone at 230 V-50 Hz (USC300T ultrasound waterbath, VWR, Fontenay-sous-Bois, France) for 2 min and rinsed with distilled water. Then, samples were disinfected with 70% ethanol and set to dry under a class 2 biosafety cabinet in sterile Petri dishes.

### 4.3. Bacterial Strains

*Staphylococcus aureus* ATCC6538, *Enterobacter aerogenes* ATCC13048 (Deutsche Sammlung für Mikroorganismen und Zellkulturen, Braunschweig, Germany) and clinical strains (Table 2) used in this study were kept at −20 °C until use.

### 4.4. Strain Preparation

Several subcultures were made in Luria Bertani broth (VWR, France) and finally on Tryptic Soy Agar (TSA) (VWR, France). Two parameters were investigated: the number of subcultures prior to the assay (two or three) and the incubation time before using the strain, i.e., 24 or 48 h, as recommended by US EPA or AFNOR guidelines [22,23].

### 4.5. Inoculum Preparation

Inocula were adjusted to Mc Farland 4 in sterile saline solution. Their purity was checked by streaking on TSA. An organic soil load was prepared with 30 g/L albumin (Merck, Fontenay-sous-Bois, France) and Triton X-100 (Merck, France) at 0.01%. It was added to the inocula (6%, V/V) to mimic the organic contamination found in droplets of saliva or on the cutaneous surface, for example. A viability test was also carried out by seeding 1 µL of the inocula in LB broth. The latter test is made to rule out a problem of bacterial viability in the inocula when no bacteria are recovered from any of the metallic surfaces tested.

### 4.6. Inoculum Deposit and Exposure

The deposit of either 1 (non-spread) or 9 µL (spread on a 359.7 mm² surface) was made on at least three samples of brass, three of 304 L stainless steel, and three of 99.95% copper. Samples were left to dry at room temperature and hygrometry. The final exposure time typically ranged from 2 to 5 min.

### 4.7. Neutralization

After drying, all samples were put in either 10 or 20 mL of Letheen broth (VWR, France). Samples were then either sonicated or shaken with glass beads for 5 min to ensure a maximal recovery of the residual bacteria. A sterility test consisting of a non-inoculated metal sample of each kind was similarly processed. At the same time, to validate the efficiency of the neutralizing solution, one sample of each metal was put in either 10 or 20 mL of Letheen broth along with 60 colony forming units (CFU) of the strain tested and similarly processed.

### 4.8. Enumeration

Decimal dilutions of Letheen broth were carried out in sterile saline from 10^−1^ to 10^−3^ and 250 µL were spread in duplicate on TSA. For neutralization and sterility assays, 250 µL of undiluted Letheen broth were inoculated on TSA. All plates were incubated for 48 h at 37 ± 1 °C before reading.

### 4.9. Filtration

To lower the detection limit for assays on clinical strains, a filtration step of the residual volume of Letheen broth on a 0.45 µm mixed cellulose esters membrane (Merck Millipore, Darmstadt, Germany) was added. The membrane was placed on TSA and incubated for 48 h at 37 ± 1 °C before reading.

### 4.10. Calculations

The results of bacterial enumerations are expressed as log CFU/metal sample and calculated using Equation (1).

(1) $$\log\left(CFU/metallic\;sample\right)=\log_{10}\left(\left(\left(CFU\;number\;count\times dilution\;factor\right)/0.25\right)\times 10\right)$$

The reduction in surviving bacteria between stainless steel 304L (negative control) and the antimicrobial surfaces (brass and copper) was calculated with Equation (2).

(2) $$\mathrm{Reduction}=100-\left(\left(\sum^{\;}Brass\;or\;copper\;enumerations\right)/\left(\sum^{\;}Stailess\;steel\;enumerations\right)\times 100\right)$$

### 4.11. Statistical Analysis

Differences between enumerations for stainless steel, brass and copper were computed with Mann–Whitney test with R software version 3.4.2 (https://www.r-project.org). A *p*-value < 0.05 was considered as significant.

### 4.12. Data Availability

The results used to prepare this paper can be accessed online through the following link: https://osf.io/ersbc/?view_only=9489ebbb47024e70bebc0c49847ff504.

## 5. Conclusions

A WCS protocol for testing the antibacterial effect of non-porous surfaces in conditions close to those found in healthcare facilities was validated on one Gram-positive and one Gram-negative reference strain. Using this protocol, the measured antibacterial efficiency of a brass alloy against 10 antibiotic-resistant strains of Gram-positive and Gram-negative bacteria representative of the main players in HAIs was found to meet existing standards for non-porous antimicrobial surfaces after a 5-min exposure at ambient temperature and hygrometry. To ensure an optimal efficiency of this antibacterial surface and a proper use in healthcare facilities, further work is now needed to (i) check that this antibacterial efficiency is retained with a longer period of exposure (e.g., 120 min instead of 5 min), (ii) verify the long-term effectiveness of brass using aged brass samples, and (iii) assess the impact of oxidation processes induced by detergents/disinfectants on the antibacterial efficiency of the surface.

## Figures and Tables

**Table 1:** Comparison of the various parameters tested for the validation of the worst case scenario protocol.

Tested Parameters		Inoculum Count (log_10_)	Recovery on Brass (log_10_)
**Inocula**	2S24H ^a^	9.5 ± 0.17 ^b^	4.4 ± 0.85
	2S48H	9.4 ± 0.13	4.5 ± 0.66 **
	3S24H	9.4 ± 0.13	3.7 ± 1.26 *
	3S48H	9.3 ± 0.11	2.8 ± 1.24 *^,^**
**Deposit**	9 µL, spread	6.0 ± 0.20	1.9 ± 0 **
	1 µL, non-spread	6.1 ± 0.11	2.74 ± 1.06 **
**Recovery Volume (Letheen Broth)**	10 mL	6.1 ± 0.09	0.7 ± 1.28
	20 mL	6.3 ± 0.10	0.9 ± 1.57
**Recovery Technique**	Ultrasonication	6.1 ± 0.02	2.3 ± 0.61
	Glass beads	6.1 ± 0.02	2.0 ± 0.61

^a^ Conditions tested for the preparation of bacteria were two subcultures with a 24 h-incubation at 37 °C (2S24H), two subcultures with a 48 h-incubation at 37 °C (2S48H), three subcultures with a 24 h- incubation at 37 °C (3S24H), three subcultures with a 48 h-incubation at 37 °C (3S48H). ^b^ Results expressed as mean ± SD. Results statistically different (Mann–Whitney test) for two given conditions of the same tested parameters at * *p* < 0.05, ** *p* < 0.001.

**Table 2:** Characteristics of the bacterial strains tested.

Strain Number	Bacterial Species	Resistance Mechanisms	Sampling Origin	Sampling Year
**ABAM14**	*Acinetobacter baumannii*	*Oxa-23, AmpC,* TEM	Rectal	2016
**ABAM41**	*Acinetobacter baumannii*	*Oxa-23, AmpC, ArmA*	Environment	2017
**EFUMAM2**	*Enteroccus faecium*	*VanA*	Rectal	2017
**EFISAM2**	*Enterococcus faecalis*	*VanB*	Rectal	2014
**ECLOAM1**	*Enterobacter cloacae*	Carbapenemase (*Oxa-48*) Extended-spectrum β-lactamase	External Quality Control	2019
**ECOLAM1**	*Escherichia coli*	Extended-spectrum β-lactamase	Rectal	2019
**KPNAM1**	*Klebsiella pneumoniae*	Extended-spectrum β-lactamase	Rectal	2019
**KPNAM2**	*Klebsiella pneumoniae*	Carbapenemase (*KPC*)	Rectal	2019
**AM85**	*Pseudomonas aeruginosa*	Overexpression of efflux pump	Sputum	2008
**PAAM10**	*Pseudomonas aeruginosa*	Carbapenemase (*VIM*)	Colostomy	2017
**SAAM33**	*Staphylococcus aureus*	*MecA,* Overexpression of efflux pump	Tracheal	2012
**SAAM118**	*Staphylococcus aureus*	*MecA*	Nasal	2019

**Table 3:** Efficacy of the copper alloy on the 12 antibiotic-resistant clinical strains tested according to the chosen worst-case scenario.

Strain	CFU/Sample (log_10_)			Reduction (%)	
	Stainless Steel	Brass	Copper	Brass/Stainless Steel	Copper/Stainless Steel
**ABAM41**	5.1 ± 5.20 ^a^	1.2 ± 1.43	3.8 ± 3.99	99.95 ± 0.051 ^a,^*	93.15 ± 11.517 *
**ABAM14**	0	0	0	ND ^b^	ND
**EFISAM2**	0	0	0	ND	ND
**EFUMAM2**	3.0 ± 3.31	−0.1 ± 0.30	3.1 ± 3.42	99.92 ± 0.010 *^,†^	60.66 ± 53.297 **^,†^
**ECLOAM1**	5.0 ± 4.83	2.2 ± 2.67	2.1 ± 2.41	99.44 ± 0.913 *^,†^	99.73 ± 0.342 *^,†^
**ECOLAM1**	4.9 ± 4.93	1.5 ± 1.89	3.9 ± 4.26	99.34 ± 0.373 *^,†^	89.13 ± 3.093 *^,†^
**KPNAM2**	5.0 ± 5.01	1.8 ± 2.20	1.9 ± 2.18	99.16 ± 0.582 *	98.03 ± 2.343 *
**KPNAM1**	3.8 ± 3.86	1.1 ± 1.34	1.8 ± 2.11	99.77 ± 0.160 *	99.95 ± 0.068 *
**AM85**	4.7 ± 4.82	0	−0.5 ± −0.01	100.00± 0 *	99.95 ± 0.094 *
**PAAM10**	4.6 ± 5.01	−0.5 ± −0.34	−0.5 ± −0.16	99.97 ± 0.043 *	100.00 ± 0.001 *
**SAAM33**	5.2 ± 5.54	2.3 ± 2.45	2.0 ± 2.34	99.85 ± 0.129 *^,‡^	99.97 ± 0.053 *^,‡^
**SAAM118**	5.1 ± 5.24	2.7 ± 3.04	2.3 ± 2.67	99.63 ± 0.524 *	99.91 ± 0.061 *

^a^ results expressed as mean ± SD. ^b^ ND: not determined. For these strains, no bacteria could be retrieved from the stainless steel surface. The calculations were therefore not possible. Statistically significant reduction of the inoculum compared to stainless steel (Mann–Whitney test) at * *p* ≤ 0.0001, ** *p* ≤ 0.05. Statistically significant difference between copper and brass reductions (Mann–Whitney test) at ^†^ *p* ≤ 0.001, ^‡^ *p* ≤ 0.05.
